# A collector-generator cell for in-situ detection of electrochemically produced H_2_

**DOI:** 10.1016/j.heliyon.2024.e27009

**Published:** 2024-02-29

**Authors:** Ling Fei, Degao Wang

**Affiliations:** a Engineering Laboratory of Advanced Energy Materials, Ningbo Institute of Materials Technology and Engineering, Chinese Academy of Sciences, Ningbo, Zhejiang, 315201, PR China; b University of Chinese Academy of Science, Beijing, 100049, PR China; c Research Center for Advanced Interdisciplinary Science of Ningbo Material Institute, Ningbo, Zhejiang, 315201, PR China

**Keywords:** Electrocatalysis, Collector-generator cell, Water splitting, H_2_

## Abstract

A collector-generator (C-G) cell used for the in-situ detection of H_2_ generated through electrochemical catalysis was described. The cell was mainly assembled with two fluorine-doped tin oxide (FTO) electrodes deposited with Pt nanoparticles, the magnitude of the current generated by the electrocatalytic oxidation of H_2_ at the collector was used for quantitative analysis of H_2_ generated at the generator. When the generator potential was set at −0.5 ∼ −0.6 V *vs.* Ag/AgCl and the collector potential at 0.4 ∼ 0.5 V *vs.* Ag/AgCl, the total Faradaic efficiency of the C-G cell could stabilize about 70%, the detection limit was about 45 μmol/L and the sensitivity was about 1 mA/55 μmol L^−1^. This dual working electrode technology could provide a convenient and rapid method for H_2_ determination and evaluate the performance of H_2_ generation catalysts that assembled on the semiconductor thin-films.

## Highlights

• The collector-generator cell can be used for in-situ detection of H_2_ in weak acid electrolyte.
• The collector-generator cell technology was proved to be rapid and stable.
• The total Faradaic efficiency of the collector-generator cell can stabilize about 70% ± 10%.

## Graphical abstract

The in-situ detection of H_2_ in the Collector-Generator (C-G) cell device is realized by oxidation of H_2_ generated at the generator to H^+^ at the collector.

**Figure:**
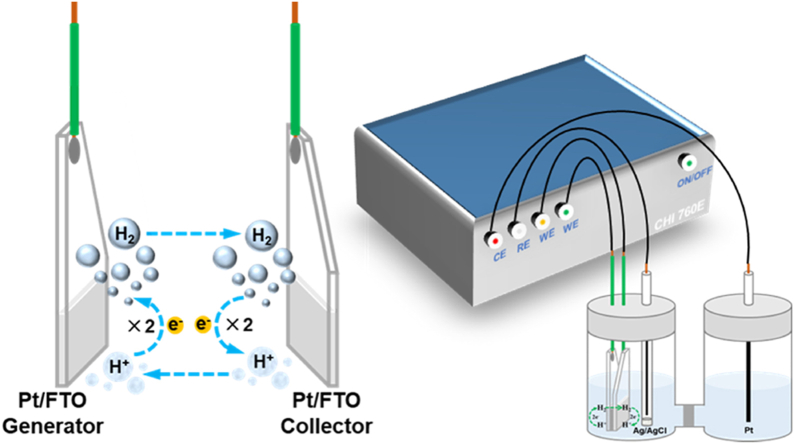

## 1 Introduction

Excessive extraction and utilization of fossil fuels have led to a global energy crisis [1,2]. Therefore, the world is urgently seeking and developing clean, and efficient renewable energy sources. Hydrogen, as an alternative energy, has been widely applied in energy storage, industry and transportation [[3], [4], [5]]. Its main advantages include: hydrogen has a higher energy density (120 MJ/kg) compared to traditional fuels [6], and it can be converted into electricity through water electrolysis and fuel cell technology.

Hydrogen can be obtained from fossil fuels or renewable resources. Among them, electrochemical water splitting is one of the simplest methods to produce H_2_ [[7], [8], [9]], and photoelectrochemical (PEC) water splitting is also a promising technology to obtain renewable fuel [[10], [11], [12], [13], [14]]. As for these strategies, H_2_ production catalyst plays a crucial role. Its fundamental function is to reduce the activation energy of the water-splitting, increasing the reaction rate. With the extensive concern over environment impacts, developing low cost, high activity and durable catalysts has become particularly important. During these catalytic reaction process, determining the H_2_ concentration is an intuitive method to evaluate the activity of catalysts. Currently, the most widely used quantitative detection of H_2_ are gas chromatography and electrochemical hydrogen gas sensing method.

Gas chromatography has the advantages of fast analysis speed and high sensitivity, with a detection limit of up to μmol/L. However, gas chromatography often requires a relatively large sample size, which may not be ideal for applications where only small amounts of H_2_ are available.

An electrochemical hydrogen gas sensor operates by detecting the electrochemical reactions that occur when H_2_ interacts with its electrodes [15,16]. It can measure real-time changes of H_2_ concentration in the reaction system. However, it requires regular calibration to ensure accurate and reliable measurements, which can be time-consuming and require additional resources.

The previously reported collector-generator (C-G) cell technology, which is based on the electrochemical reaction mechanism, can simultaneously perform electro-oxidation and electro-reduction reactions. With the advantages of convenience, accuracy and reproducibly, it has been applied in the detection of oxygen production through electrochemical catalysis in some neutral solution systems [17,18].

**Fig. 1:**
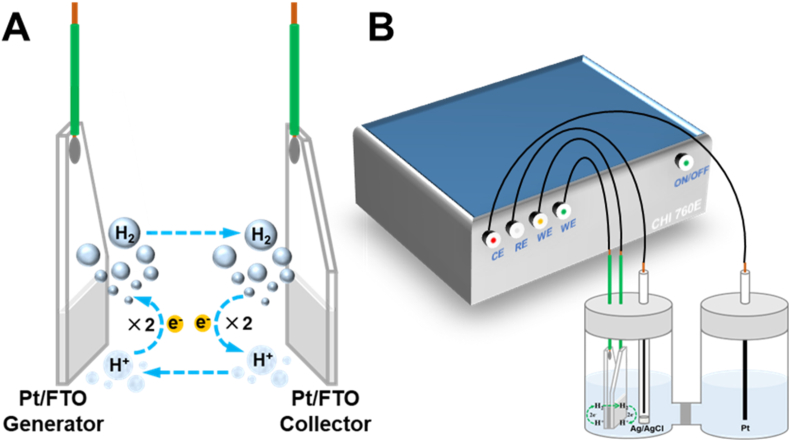
**(A)** The collector-generator (C-G) cell schematic; **(B)** The three-electrode system assembled in an H-typed glass electrolytic cell.

In this work, we constructed a C-G cell using fluorine-doped tin oxide (FTO) electrodes deposited with Pt nanoparticles on the conductive surfaces to achieve in-situ detection of electrocatalytic H_2_ production (Fig. 1). The purpose of using a Pt/FTO generator here was to provide a simple method to generate H_2_. As shown in Fig. 1A, by applying a constant reduction potential, H^+^ at the generator was reduced to H_2_, which rapidly diffused to the collector. At the collector, H_2_ was oxidized back to H^+^ with a constant oxidation potential. Compared to the bulk solution, the concentration of H_2_ increased more rapidly between the generator and the collector. Thus, the magnitude of the current generated by the electrocatalytic oxidation of H_2_ at the collector could be used for quantitative analysis of H_2_ generated at the generator. Based on the preceding mechanism of H_2_ redox reaction, the C-G cell has the potential to become a general approach to evaluate the performance of H_2_ generation catalysts that assembled on the semiconductor thin-films.

## 2 Experimental section

### 2.1 Fabrication of the experimental apparatus

In this study, both the generator and the collector were prepared by thermal decomposition of reducing chloroplatinic acid to Pt nanoparticles deposited on FTO. The solution of 10% chloroplatinic acid in isopropanol was doctor-bladed in 1 cm wide area on a thoroughly cleaned FTO glass slide. After air drying, the FTO was placed in a muffle furnace and heated at a ramp rate of 10 °C/min to 385 °C, and held for 30 min before cooling to room temperature. The surface morphology of Pt/FTO was characterized by using thermal field emission (TFE) scanning electron microscopy (SEM) image and energy dispersive X-ray spectroscopy (EDS). The SEM image in Fig. S1 showed the Pt nanoparticles uniformly distributed on the FTO substrate.

The C-G cell was assembled using two identical Pt/FTO electrodes (1 cm × 4 cm). The top right corner of each Pt/FTO electrode's conductive surface was removed, and a wire was bonded using silver conductive adhesive to the top left corner. On each side of one Pt/FTO electrode, a 1 cm long, 1 mm wide, and 1 mm thick insulating glass slide was bonded using inert epoxy resin, and it was bonded facing the conductive surface of the other Pt/FTO electrode. The distance between the two Pt/FTO electrodes was approximately 1 mm.

### 2.2 Electrochemical experiment

The entire electrochemical reaction device mainly consisted of the C-G cell, the Ag/AgCl reference electrode and the platinum wire counter electrode, which was used to accurately quantify the oxidation current of the collector electrode and the reduction current of the generator (Fig. 1B). The entire device was placed in an H-type glass electrolysis cell, with a portion of the C-G cell immersed in the electrolyte solution, as shown in Fig. S2. Each side of the device was filled with 0.1 M acetate buffer solution at pH 4.56 in 0.5 M NaNO_3_. The addition of NaNO_3_ as the supporting electrolyte could improve the ionic strength and the conductivity of the acetate buffer solution, which was particularly important for achieving stable and reproducible measurements. Both sides of the H-type cell were sealed with PTFE plug, and all the electrochemical experiments were conducted using a CH-Instruments 760E bipotentiostat.

### 2.3 Data analysis

The mole of H_2_ generated at the generator or oxidized at the collector was estimated according to the following Faraday's law [19], assuming that the electron count was 2 per molecule H_2_ at the generator:

(1) $$i_{\text{gen}/\text{col}}t=zFn_{\text{gen}/\text{col}}$$

In Eq. (1), $i_{\text{gen}/\text{col}}$ was the current at each electrode during the reaction, $n_{\text{gen}/\text{col}}$ was the mole of H_2_ at the generator or collector, and *F* is Faraday constant.

The diffusion loss of H_2_ was measured by using gas chromatography, and the mole of H_2_ loss was calculated through the following standard gas unit conversion formula, which was then substituted into Eq. (2):

(2) $$C_{H_{2}}\left(\text{mg}/m^{3}\right)=M\;/\;22.4\;C_{H_{2}}\left(\text{ppm}\right)$$

(3) $$n_{\text{loss}}=10\;\exp\;-9V_{\text{loss}}\;C_{H_{2}}\left(\text{mg}/m^{3}\right)\;/\;M_{H_{2}}$$

In Eq. (2), $C_{H_{2}}$ was the concentration of H_2_ loss in the space above the solution on at the C-G cell compartment, $M_{H_{2}}$ was the molar mass of H_2_. In Eq. (3), $n_{\text{loss}}$ was the molar quantity of H_2_ loss, and $V_{\text{loss}}$ was the volume of the space above the solution at the C-G cell compartment.

The collection efficiency of the collector could be determined by Eq. (4). The total Faradaic efficiency of the C-G cell could be determined by Eq. (5), assuming that all the lost H_2_ diffused into the space above the solution:

(4) $$\eta_{\text{col}}=n_{\text{col}}\;/\left(n_{\text{col}}+n_{\text{loss}}\right)$$

(5) $$\eta =n_{\text{col}}\;/\left(n_{\text{gen}}\;\eta_{\text{col}}\right)$$

In Eq. (4), $\eta_{\text{col}}$ was the collection efficiency of the collector. In Eq. (5), η was the total Faradaic efficiency of the C-G cell.

Since the distance between the two electrodes dictated the time delay for H_2_ diffusion from the generator to the collector, the influence of different distances on the collector efficiency were compared. As shown in Fig. S3, with the increase of the distance, the collector efficiency decreased obviously, Thus, the distance between the two Pt/FTO electrode was fixed to 1 mm.

## 3 Results and discussion

### 3.1 Electrochemical performances of the C-G cell

**Fig. 2:**
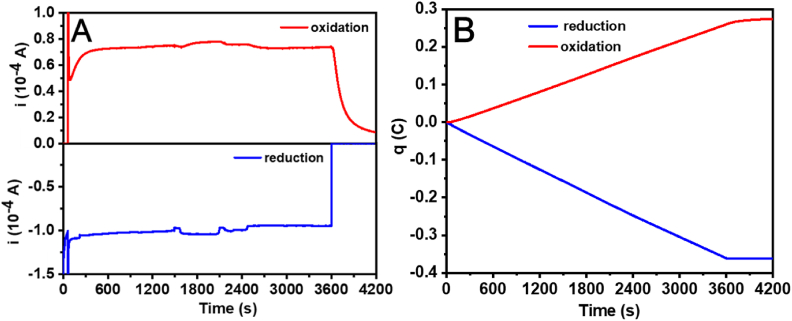
**(A)** The current-time curve of the collector (Top) and the current-time curve of the generator (Bottom). **(B)** The charge-time curve of the C-G cell. The red line represents the charge passed through the generator, and the blue line represents the charge passed through the collector.

Fig. 2A showed the current response of the C-G cell during the reaction. Prior to the reaction, the electrolyte solution was saturated with Ar_2_ to ensure the absence of O_2_ in the sealed microreactor. Using a 1 mm distance between the electrodes gave a characteristic time of about 60 s to reach a steady-state reduction current, and which could be used to determine the true Faradaic efficiency of the C-G cell [11]. Thus, within the first 60 s, the generator potential was maintained at −0.5 V *vs.* Ag/AgCl to generate H_2_. Then, the generator potential was held constantly while the collector potential was applied at 0.5 V *vs.* Ag/AgCl, oxidizing the diffused H_2_ back to H^+^. After 3600 s, the applied potential at the generator was switched to 0 V *vs.* Ag/AgCl, ceasing the evolution of any H_2_. Meanwhile, the potential at the collector was maintained at 0.5 V *vs.* Ag/AgCl to oxidize the residual H_2_ to H^+^, ensuring a full accounting of all H_2_ generated at the generator during the experimental process.

Fig. 2B recorded the integrated current curves of the C-G cell. The total charge passed through the collector was less than that of the generator, indicating a collection efficiency of less than 100%. This was primarily attributed to the diffusion loss of H_2_ in the space above the solution at the C-G cell compartment. After the electrochemical experiments, the diffusion loss of H_2_ was measured by gas chromatography to calibrate the collector efficiency. The standard working curve of H_2_ was shown in Fig. S4.

To attempt to understand the kinematic for the C-G cell, the Tafel plots were examined (Fig. S5). The Tafel equation (η = b*log *j* + *a*, where η is overpotential, *j* is the current density, *b* is the Tafel slope, and *a* is the Tafel constant) was applied. The rate determining step for the generator was suggested to be the Volmer reaction (Tafel slope ∼108 mV/decade) and the Tafel slope for the collector was ∼726 mV/decade.

**Fig. 3:**
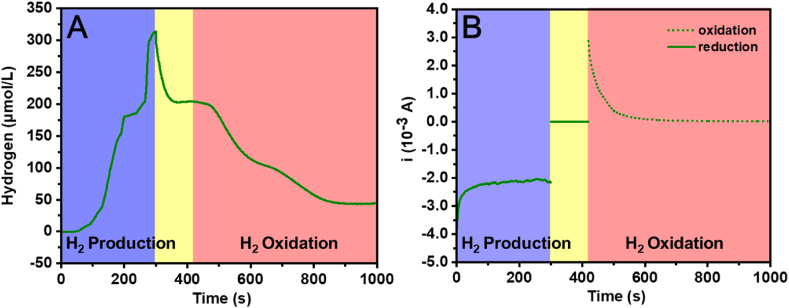
**(A)** The H_2_ concentration measured by hydrogen microsensor. **(B)** The corresponding current-time curves of the generator (solid line) and the collector (broken line) of the C-G cell.

To evaluate the detection limit and the sensitivity of the C-G cell, Unisense H_2_-NP needle microsensor was located at the dual working electrode compartment to monitor the change of H_2_ concentration. As shown in Fig. 3A, the H_2_ concentration reached about 313 μmol/L within 5 min with the generator potential set at −0.6 V *vs.* Ag/AgCl. The sharp drop appeared subsequently was due to the diffusion of H_2_ from the generator to the bulk solution. When the H_2_ concentration stabilized at about 203 μmol/L, the collector potential was set at 0.5 V *vs.* Ag/AgCl, the decrease of the H_2_ concentration proved that H_2_ was deoxidized to H^+^, and it remained at 45 μmol/L after 8 min. During the H_2_ oxidation progress, the oxidation current decreased from 2.87 mA to 0.01 mA, shown as Fig. 3B. According to the data, the detection limit was about 45 μmol/L and the sensitivity was about 1 mA/55 μmol L^−1^ for the C-G cell. Table S1 displayed the comparison data of H_2_ detection methods between this work, commercial methods and reported literature [20]. The results demonstrated the potential viability of our C-G cell for rapid detection of H_2_.

In addition, we also tested the stability of the C-G cell. The electrochemical experiment was carried out with the generator potential held at −0.6 V *vs.* Ag/AgCl and the collector potential at 0.5 V *vs.* Ag/AgCl. As shown in Fig. S6A, the C-G cell performed stably for 15 h with a little fluctuation less than 0.01 mA, and it also showed no significant change in the CV over 500 cycles (Fig. S6B).

### 3.2 Influence of applied potential, supporting electrolyte and buffer solution pH

**Fig. 4:**
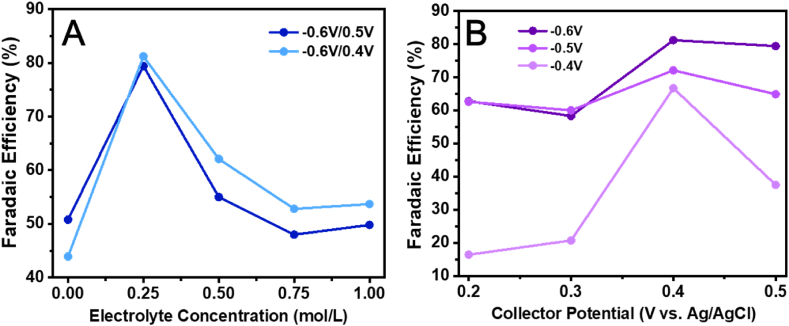
The total Faradaic efficiency of the C-G cell vs **(A)** electrolyte concentration and **(B)** collector potential in 0.1 M acetate buffer at pH 4.56.

To investigate the influence of experimental factors on the total Faradaic efficiency of the C-G cell, the effects of applied potential, supporting electrolyte concentration, and buffer solution pH were discussed. The generator potential was set at −0.4 V ∼ −0.6 V *vs.* Ag/AgCl, while the collector potential was set at 0.2 V ∼ 0.5 V *vs.* Ag/AgCl. In these experiments, the concentration of NaNO_3_ in the buffer solution (0.1 M acetate buffer at pH 4.56) was 0 mol/L ∼ 1.0 mol/L. As shown in Fig. 4, both the supporting electrolyte concentration and applied potential affected the total Faradaic efficiency of the C-G cell, with a higher efficiency observed at low electrolyte concentrations and lower efficiency at the higher electrolyte concentrations. When the generator potential was set at −0.5 V ∼ −0.6 V *vs.* Ag/AgCl and the collector potential at 0.4 V ∼ 0.5 V *vs.* Ag/AgCl, a total Faradaic efficiency of 70% ± 10% was observed. In addition, the C-G cell could maintain stable performance in buffer solution at different pH (Fig. S7).

Determining the accuracy of the C-G cell has major importance for ensuring the Faradaic efficiency reliably and reproducibly. As for our device, the accuracy was the collection efficiency of the collector. Thus, four independent experiments were carried out with the generator held at −0.5 ∼ −0.6 V *vs.* Ag/AgCl to form H_2_ and the collector potential at 0.4 ∼ 0.5 V *vs.* Ag/AgCl to oxide H_2_. From the data listed in Table S2, the average collection efficiency was about 85% ± 5%, which could support the reliability and repeatability of the C-G cell.

## 4 Conclusions

Our work described a C-G cell design for in-situ detection of electrochemical H_2_ production. The C-G cell consisted of two FTO electrodes deposited with Pt particles. The distance between the two Pt/FTO electrodes was 1 mm, allowing for the diffusion of H_2_ from the generator to the collector, where it was rapidly oxidized to H^+^. The experimental results demonstrated that such a C-G cell offered advantages with reproducibly and reliably for H_2_ determination.

## Data availability

Data included in article/supp. material/referenced in article.

## CRediT authorship contribution statement

**Ling Fei:** Writing – original draft, Methodology, Formal analysis, Data curation, Conceptualization. **Degao Wang:** Writing – review & editing, Supervision, Resources, Methodology, Conceptualization.

## Declaration of competing interest

The authors declare that they have no known competing financial interests or personal relationships that could have appeared to influence the work reported in this paper.
