# Supplementary material for: A collector-generator cell for in-situ detection of electrochemically produced H2
Source: Heliyon. 2024 Feb 29;10(5):e27009. doi: 10.1016/j.heliyon.2024.e27009 (PMC10915372; doi:10.1016/j.heliyon.2024.e27009)
Supplement: Multimedia component 1 [file mmc1.docx]

Supporting Information

A Collector-Generator Cell for In-situ Detection of Electrochemically Produced H_2_

*Ling Fei^a^, Degao Wang^a,b,c,^**

^a^ Engineering Laboratory of Advanced Energy Materials, Ningbo Institute of Materials Technology and Engineering, Chinese Academy of Sciences, Ningbo, Zhejiang 315201, P. R. China.

^b^ University of Chinese Academy of Science, Beijing, 100049, P. R. China.

^c^ Research Center for Advanced Interdisciplinary Science of Ningbo Material Institute, Ningbo, Zhejiang 315201, P. R. China.

*Corresponding author

* (D.G. Wang) E-mail address: wangdegao@nimte.ac.cn.

**Experimental Section**

**Reagents and Materials**

All chemicals used including chloroplatinic acid hexahydrate (Pt ≥37.5%), isopropanol (≥99.8%), acetic acid (≥99.5%), sodium acetate anhydrous (≥99.0%), sodium nitrate (≥99.0%), sodium perchlorate (≥98.0%) and sodium sulfate anhydrous (≥99.0%) were obtained from Aladdin. FTO ( 14 Ω/sq) was purchased from Jinge.

[**Gas Chromatography**](javascript:;)

The diffusion loss of H_2_ was quantitatively measured using gas chromatography to calibrate the collection efficiency. At the end of an electrochemical experiment, 0.5 mL of a gaseous sample was extracted from above the solution on the side where the C-G cell was placed using a micro syringe. The gas sample was then injected into a gas chromatograph (Agilent 8890 GC system) for quantitative analysis of the H_2_ escaped from the generator to calibrate the collection efficiency of the device. The flow rate of GC carrier gas Ar_2_ was maintained at 34.0 mL/min, and the inlet temperature was maintained at 150 ℃. The ion source temperature of the thermal conductivity detector (TCD) was kept at 200 ℃. The chromatographic diagram of H_2_ was displayed below in **Figure S3**. The retention time of H_2_ was 150 seconds. The standard working curve of H_2_ was established based on different concentration gradients, with a correlation coefficient r^2^ of 0.995.

**H_2_ Detection Experiments**

Hydrogen evolution generated during the electrochemical measurement was monitored using Unisense H_2_-NP needle microsensor located at the dual-working electrode compartment. Before the experiment, the calibration of the hydrogen microsensor was carried out. The sensitivity was determined according to the current passed on the collector during the electrochemical measurement and the total amount of generated H_2_ on the generator.

**
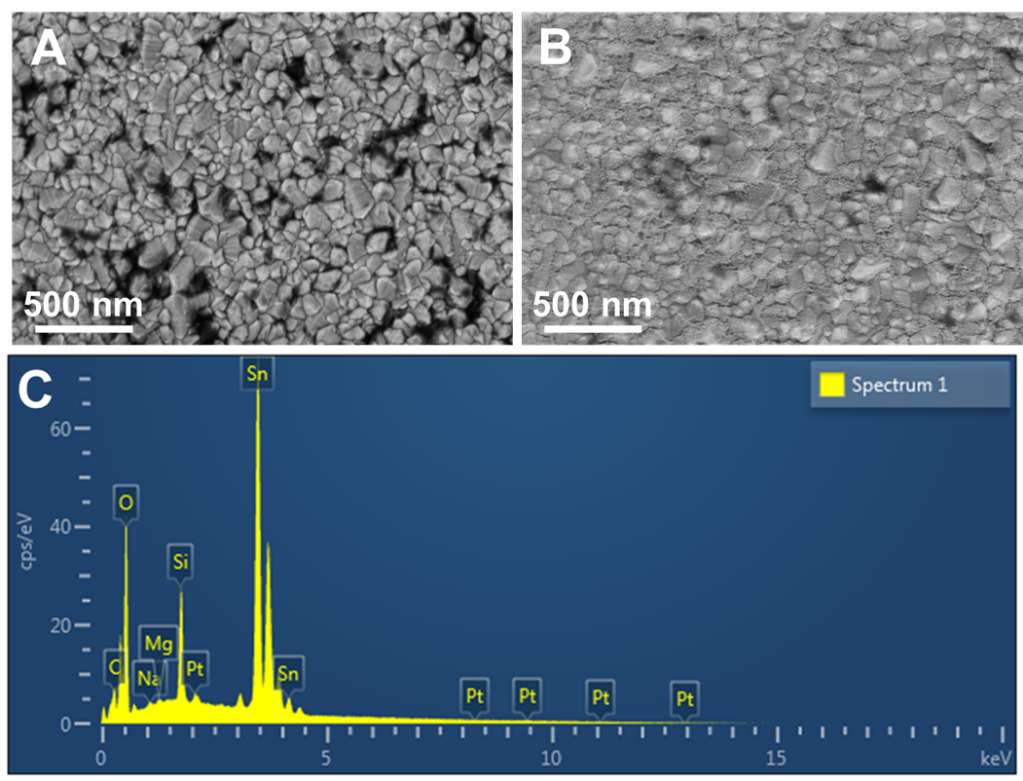
**

**Figure S1.** SEM image of FTO **(A)** and Pt/FTO **(B)**, EDS of Pt/FTO **(C)**.


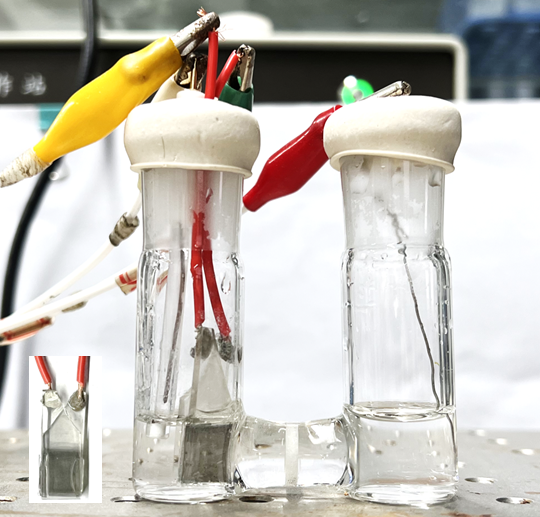


**Figure S2.** The photographs of the C-G cell consisting of Pt/FTO electrodes and the three-electrode system assembled in an H-typed glass electrolytic cell.

**Figure S3.** The collector efficiency vs distance between two Pt/FTO electrodes.


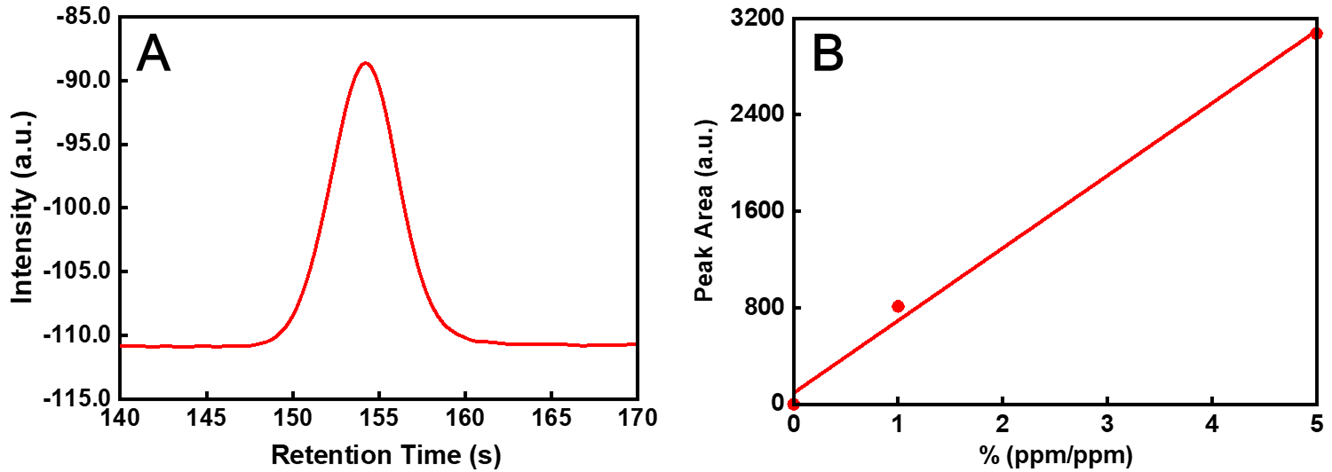


**Figure S4.** Chromatogram of H_2_ using GC and the standard working curve.


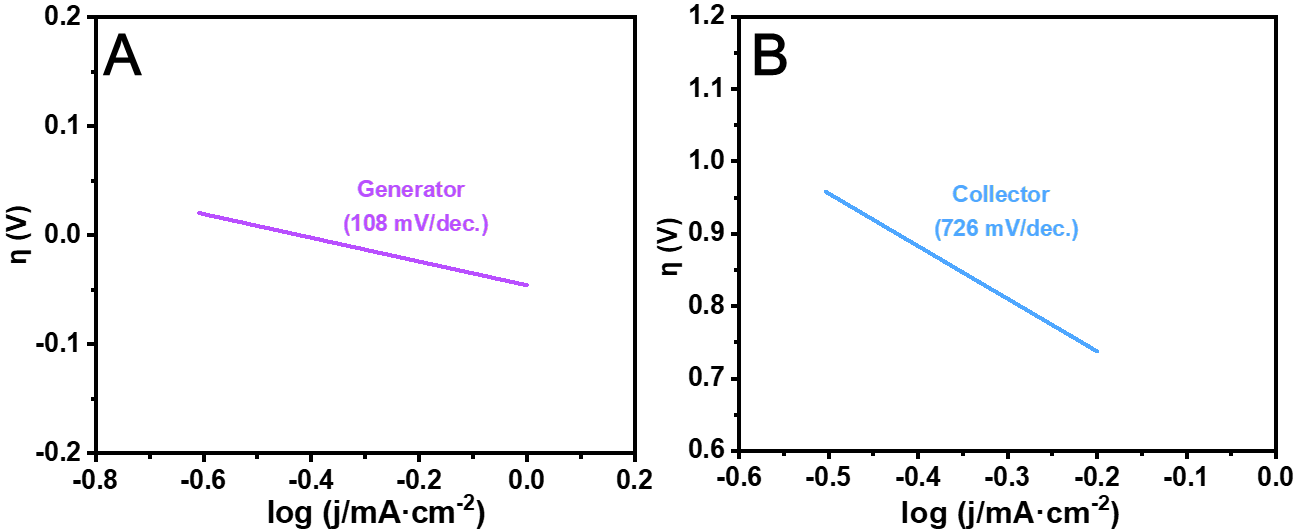


**Figure S5**. Tafel plot of **(A)** the generator and **(B)** the collector taken at 25 mV/s.

**
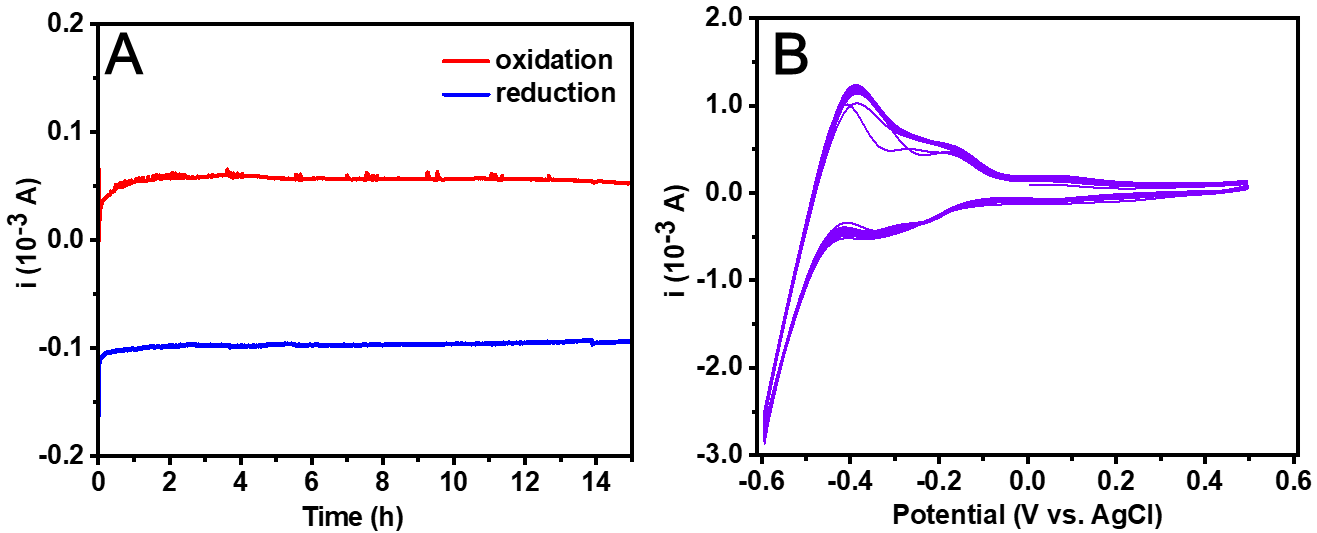
**

**Figure S6**. **(A)** Current and **(B)** CV of the C-G cell in 0.1 M acetate buffer at pH 4.56 in 0.25 M NaNO_3_. Scan rate 50 mV/s.

**Figure S7.** The total Faradaic efficiency vs the buffer pH (0.1 M HClO_4_ buffer at pH 1.0 in 0.25 M NaNO_3_, 0.1 M acetate buffer at pH 3.0, 4.56, 5.6 in 0.25 M NaNO_3_).

**Table S1 Comparison of H_2_ detection methods**

| Detection method | Sensitivity | Detection limit  (μmol/L) |
| --- | --- | --- |
| Collector-generator Cell  (This work) | 1 mA / 55 μmol·L^-1^ | 45 |
| Gas Chromatography  (Agilent 8890) | — | 2 |
| Microsensor  (Unisense H_2_-NP ) | 1 mV / 2 μmol·L^-1^ | 0.3 |
| Pd-Pt nanowires | nr^a^ | 22 |
| Pd-Ni nanowires | nr | 45 |
| Pd-Ti | nr | 312 |
| Pd-Co nanowires | nr | 45 |

nr^a^ = not reported

**Table S2 Electrochemical Characterization of the C-G Cell**

| Potential  (V) | $\text{n}_{\text{gen}}$  (ppm) | $\text{n}_{\text{col}}$  (ppm) | $\text{n}_{\text{loss}}$  (ppm) | $\text{η}_{\text{col}}$  (%) | $\text{η}$  (%) |
| --- | --- | --- | --- | --- | --- |
| -0.6/0.5 | 4.55 | 3.11 | 0.5 | 86.0 | 79.4 |
| -0.5/0.5 | 1.64 | 0.91 | 0.16 | 85.3 | 64.9 |
| -0.6/0.4 | 6.25 | 4.60 | 0.51 | 90.0 | 81.7 |
| -0.5/0.4 | 1.98 | 1.31 | 0.12 | 91.9 | 72.1 |
